# Diagnostic Performance of CLEIA Versus FEIA for KL‐6 Peripheral and Alveolar Concentrations in Fibrotic Interstitial Lung Diseases: A Multicentre Study

**DOI:** 10.1002/jcla.25108

**Published:** 2024-09-25

**Authors:** Miriana d'Alessandro, Sara Gangi, Irene Paggi, Piera Soccio, Laura Bergantini, Tommaso Pianigiani, Giusy Montuori, Giorgia Moriondo, Giulio Natalello, Sofia Marrucci, Alfonso Brogna, Giulia Scioscia, Donato Lacedonia, Paolo Cameli, Elena Bargagli

**Affiliations:** ^1^ Respiratory Diseases Unit, Department of Medical and Surgical Sciences & Neuro‐Sciences University of Siena Siena Italy; ^2^ Department of Medical and Surgical Sciences University of Foggia Foggia Italy; ^3^ Institute of Respiratory Diseases Policlinico Riuniti of Foggia Foggia Italy

**Keywords:** bronchoalveolar lavage, diagnosis, interstitial lung diseases, Krebs von den Lungen‐6, pulmonary fibrosis

## Abstract

**Background:**

Interstitial lung diseases (ILD) is a group of lung disorders characterized by interstitial lung thickening due to inflammatory and fibrotic processes. Krebs von den Lungen‐6 (KL‐6) is a molecule secreted by damaged type II alveolar pneumocytes in the alveolar space. The goal of the present study was to compare two detection methods of KL‐6 in both bronchoalveolar lavage (BAL) and serum from ILD patients at the moment of diagnosis.

**Methods:**

Patients with suspicious of ILD and followed at two Italian referral centres for rare lung diseases were included in the study. BAL fluid and serum were collected and analysed by chemiluminescent enzyme immunoassay (CLEIA) and fluorescent enzyme immunoassay (FEIA) methods provided by Tosoh Biosciences.

**Results:**

A total of 158 (mean age ± standard deviation, 61.5 ± 13.7, 65 females) patients were enrolled. A total of, 36 had diagnosis of idiopathic pulmonary fibrosis (IPF), 74 sarcoidosis, 15 connective tissue disease‐ILD (CTD‐ILD) and 33 other ILD. Diagnostic agreement between two methods was demonstrated for both BAL (*r* = 0.707, *p* < 0.0001) and serum (*r* = 0.816, *p* < 0.0001). BAL KL‐6 values were lower than serum (*p* < 0.0001). IPF patients had higher serum KL‐6 concentration than other ILDs (*p* = 0.0294), while BAL KL‐6 values were lower in IPF than in non‐IPF (*p* = 0.0023).

**Conclusion:**

This study explored KL‐6 concentrations through the CLEIA method in serum and BAL of patients with various ILDs, showing significant differences of biomarkers concentrations between IPF and other non‐IPF ILDs. Our findings are promising as they provided further knowledge concerning KL‐6 expression across different ILDs and may suggest its utility in differential diagnosis.

## Graphical abstract

Flowchart of the study design from the enrolment of ILD patients in the two ILD referral centres at Siena and Foggia University for collection and processing of serum and BAL samples for KL‐6 analysis through the two methods FEIA and CLEIA. BALF, bronchoalveolar lavage fluid; CLEIA, AIA‐CL300 based on chemiluminescent enzyme immunoassay; FEIA, AIA‐360 based on fluorescent enzyme immunoassay; ILD, interstitial lung diseases; KL‐6, Krebs von den Lungen‐6.

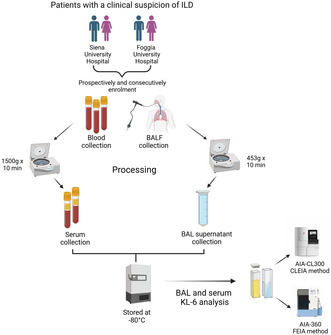

## 1 Background

Interstitial lung diseases (ILDs) are a heterogeneous group of lung disorders characterized by inflammatory and fibrotic changes in the lung interstitium [1]. They encompass about 200 lung disorders which often have overlapping imaging findings and clinical presentations [2].

Interstitial lung diseases are identified and characterized by high‐resolution computed tomography (HRCT), bronchoscopic sampling and computed and/or surgical lung biopsy. Multidisciplinary discussion involving the pulmonologist, radiologist and pathologist has become standard practice for accurate diagnosis of ILDs and for deciding therapeutic approach [1, 3].

Interstitial lung diseases may have different underlying causes and different progression [4]. Since their outcome is still largely unpredictable, a reliable biomarker could help clinicians predict their course and make more informed decisions about treatment and the goals of care. Identification of a cost‐effective non‐invasive biomarker capable of gauging disease activity and progression is therefore of great interest for scientific research [5].

In 1989, Kohno et al. discovered Krebs von den Lungen‐6 (KL‐6), a high‐molecular‐weight (200 kDa) glycoprotein expressed by type II alveolar pneumocytes [6] in the alveolar space in response to cell damage and regeneration in patients with ILD [7]. The damage and altered permeability of the alveolar‐capillary membrane induced by fibrotic and inflammatory processes allows KL‐6 to pass into the blood stream [8]. The role of biomarkers in the management of interstitial lung disease: implications of biomarkers derived from type II pneumocytes. In 1999, detection of the soluble form of serum KL‐6 by chemiluminescent enzyme immunoassay (CLEIA) was introduced in routine clinical settings in Japan [9]. In the meantime it has been proposed as a diagnostic marker of fibrotic interstitial lung disease, and today it is accepted as a reliable biomarker of disease activity [10, 11]. Elevated serum concentrations of KL‐6 are related to disease progression and mortality in ILD patients [12, 13].

In order to better understand the dynamics of KL‐6 production and release into serum, KL‐6 concentrations were recently compared in serum and BAL. Disparities at local and systemic level were documented, especially in progressive ILD phenotypes. Progressive pulmonary fibrosis can lead to greater KL‐6 release into the bloodstream, confirming the prognostic relevance of serum KL‐6 in assessing ILD severity [14].

The development of reliable new immunological kits for the detection of KL‐6 may offer faster results at a lower cost. The fluorescent enzyme immunoassay (FEIA) method was recently tested in COVID‐19 patients, proving reliable in the detection of serum KL‐6. The study confirmed the reliability of FEIA and CLEIA [15] for detecting KL‐6 as a severity marker in COVID‐19 patients.

In the present study we compared the two analytical methods, FEIA and CLEIA, for detecting KL‐6 in serum and BAL, with the aim of further validating its diagnostic value in different ILDs, including idiopathic pulmonary fibrosis (IPF), connective tissue disease‐associated with interstitial lung disease (CTD‐ILD) and sarcoidosis.

## 2 Material and Methods

### 2.1 Study Population

**FIGURE 1:**
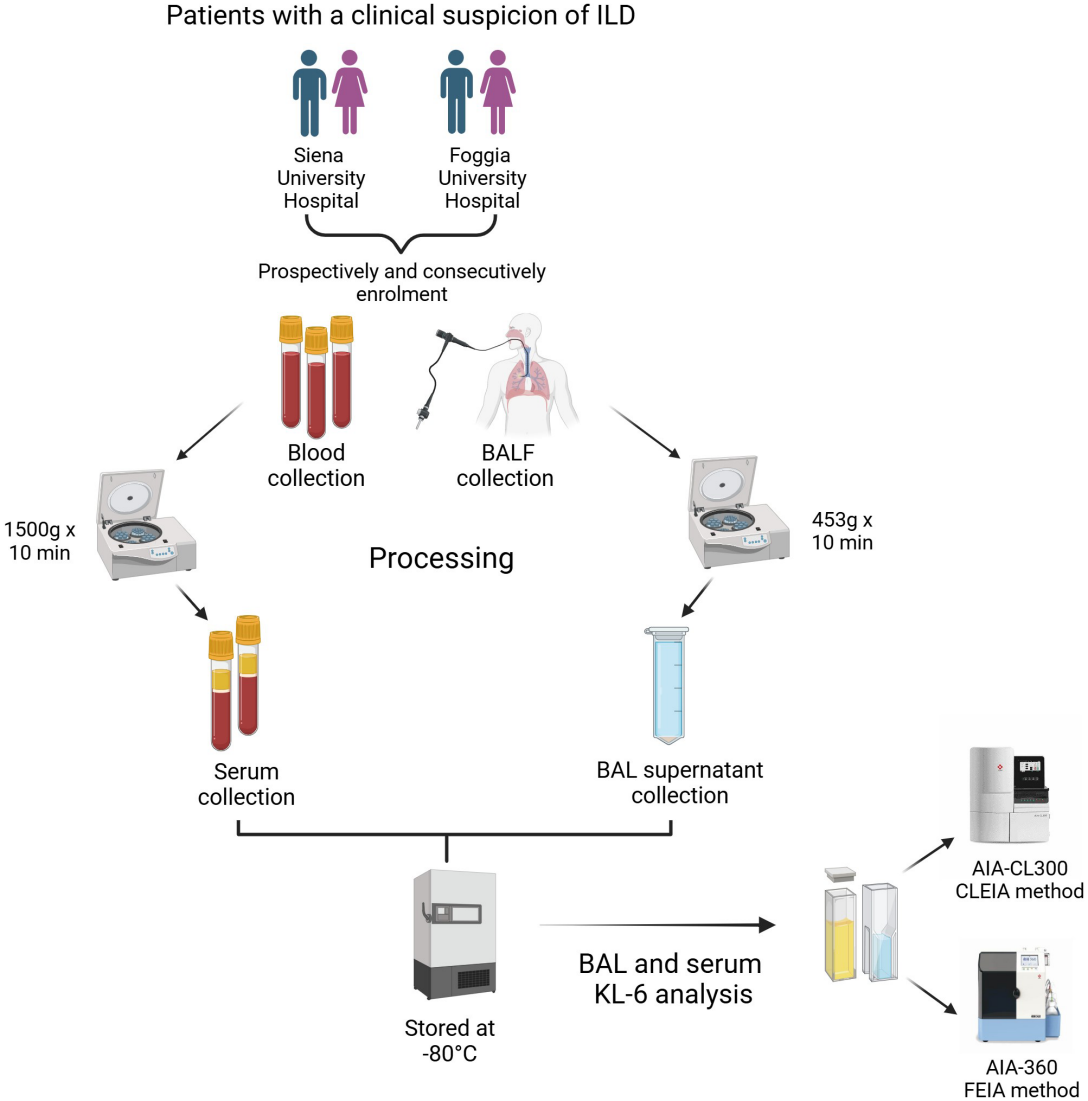
Flowchart of the study design from the enrolment of ILD patients in the two ILD referral centers at Siena and Foggia University for collection and processing of serum and BAL samples to the KL‐6 analysis through the two methods FEIA and CLEIA. BALF, bronchoalveolar lavage fluid; CLEIA, AIA‐CL300 based on chemiluminescent enzyme immunoassay; FEIA, AIA‐360 based on fluorescent enzyme immunoassay; ILD, interstitial lung diseases; KL‐6, Krebs von den Lungen‐6.

Patients with a clinical suspicion of ILD underwent a specific diagnostic pathway at the ILD referral centres of Siena and Foggia University Hospitals. The study design was summarized in Figure 1. All patients were enrolled consecutively and prospectively in the study from 2019 to 2023. An inclusion criterion was simultaneous BAL and serum sampling at the moment of bronchoscopic procedure performed for diagnostic purposes. Multidisciplinary discussion confirmed a diagnosis of IPF according to American Thoracic Society/European Respiratory Society (ATS/ERS) guidelines in 36 patients (mean age ± standard deviation, 73 ± 8 years, 6 females) [4]. Seventy‐four patients (mean age ± standard deviation, 56 ± 12 years, 33 females) were diagnosed with sarcoidosis according to international criteria based on clinical signs, chest radiography findings and non‐caseating granulomas in lymph nodes and/or endobronchial biopsy specimens [16]. All 14 patients diagnosed with CTD‐ILD (six patients with rheumatoid arthritis [17] (mean age ± standard deviation, 58 ± 17 years, five females), six with systemic sclerosis [18] (mean age ± standard deviation, 65 ± 11 years, four females) and three with Sjogren syndrome [19] (mean age ± standard deviation, 72 ± 6 years, two females)) showed a nonspecific interstitial pneumonia (NSIP) radiological pattern at chest CT scan. Thirty‐three patients had other ILDs, namely 6 had fibrotic hypersensitivity pneumonitis [4], 11 unclassifiable idiopathic interstitial pneumonia, 3 cryptogenic organizing pneumonia, 3 idiopathic NSIP [4], 2 lymphangioleiomyomatosis [4], 4 chronic eosinophilic pneumonia [4] and 4 pulmonary Langerhans cell histiocytosis [4]. The diagnosis of the specific ILD was confirmed by multidisciplinary discussion for every patient included in the study.

Bronchoscopy with bronchoalveolar lavage (BAL) was performed with written consent from the patients. Fasting blood samples and BAL fluid were collected from all patients, divided into aliquots and stored at −80°C until needed.

Demographic and clinical data, including comorbidities, family history, lung function parameters and radiological features were obtained from the medical records and entered in an electronic database for statistical analysis.

The study was approved by the regional ethical review board of Siena (C.E.A.V.S.E. Markerlung 17431) and complied with the Helsinki Declaration. All patients provided written informed consent prior to participating in the study. Healthy donors were not enrolled for ethical reasons.

### 2.2 Lung Function Test Parameters

Lung function tests were conducted on all patients, including forced expiratory volume in 1 s (FEV1), forced vital capacity (FVC) and diffusing capacity of the lungs for carbon monoxide (DLCO). The tests were carried out following ATS/ERS guidelines using a Jaeger body plethysmograph, with adjustments for temperature and atmospheric pressure [20].

### 2.3 Bronchoscopy

Patients received intravenous fentanyl (100 μg) and midazolam (3–5 mg) 15–30 min before undergoing bronchoscopy. Lidocaine was instilled to the larynx and bronchi for local anaesthesia. A Pentax bronchoscope EB15‐J10 (Pentax Medical Company, PENTAX Europe GmbH, Hamburg, Germany) was inserted through the mouth to avoid blood contamination. Physiological saline solution (3 × 50 mL) was instilled into the middle or lingual lobe. For cell analysis, 10–20 mL of pooled BAL sample was collected according to international BAL Task Force Group guidelines [21]. BAL samples were processed by filtering through sterile gauze, followed by cell count via cytocentrifuge smear (600 rpm for 5 min) using a Thermo Shandon Cytospin 3 (Marshall Scientific, Hampton, NH, USA) and stained with Diff‐Quik stain kit (DiaPath, Martinengo, BG, Italy). A total of 500 cells was counted, determining percentages of macrophages, lymphocytes and neutrophils. Cell viability was assessed by Trypan blue exclusion in a Burker Chamber. BAL samples were deemed suitable if they contained up to 5% ciliated columnar epithelial cells.

### 2.4 Flow Cytometry Analysis of Lymphocytes

Bronchoalveolar lavage samples were analysed for lymphocytes phenotyping when composed by > 5% lymphocyte differential cell counts through flow cytometry using a panel of monoclonal antibodies (BD Multitest 6‐color TBNK, San Jose, CA, USA): FITC‐labeled CD3, PE‐labeled CD16 and CD56, PerCPCy5.5‐labeled CD45, PECy7‐labeled CD4, APC‐labeled CD19 and APCCy7‐labeled CD8 according to the manufacturer's instructions. At least 50,000 events were collected for each sample. Data was analysed using DIVA software (BD‐Biosciences San Jose, CA, USA). Lymphocytes were phenotyped based on forward (FSC) versus side (SSC) scatters and additional gating was applied using SSC versus CD45 to distinguish lymphocytes from cell debris. Specific panels were subsequently assessed to identify T lymphocytes, B lymphocytes and NK cells. T lymphocyte subpopulations were gated in order to distinguish CD3^+^CD4^+^ (T‐helper), CD3^+^CD8^+^ (T‐cytotoxic) and CD3^−^ CD16^+^/56^+^ (NK) cell percentages.

### 2.5 FEIA and CLEIA

Serum and BAL concentrations of KL‐6 were determined with two AIA360 and AIA‐CL300 automatic biochemical analysers (Tosoh Biosciences) based on FEIA and CLEIA, respectively. Both instruments used the same control set with different low (L1) and high (L2) levels. FEIA recorded an ST AIA‐PACK KL‐6 L1 in the range 6.4–9.6 U/mL and L2 was in the range 42–63 U/mL. CLEIA recorded a CL AIA‐PACK KL‐6 L1 in the range 5–7.5 U/mL and L2 was in the range 30–45 U/mL.

To compare the two instruments, we measured 158 serum and 158 BAL samples from ILD patients. CLEIA was completely automated, including 1:25 dilution of serum samples and 1:10 dilution of BAL samples. FEIA required manual dilution with solution provided by the manufacturers, 1:50 for serum and 1:25 for BAL fluid.

### 2.6 Statistical Analysis

The results are reported as means ± standard deviations (SD) and medians and inter quartiles (25th and 75th percentiles) for continuous variables. The Shapiro–Wilk test showed that the data did not have a normal distribution. A Pearson's correlation test was performed to determine the correlation between the two immunoassays. Bland–Altman analysis was performed to assess agreement and bias between systems.

Multiple comparisons were assessed by non‐parametric one‐way ANOVA (Kruskal‐Wallis test) and Dunn's test. Comparison of two groups was performed by Mann–Whitney *U*‐test. The Chi‐square test was used for categorical variables. Correlations between immunological and clinical features were investigated by Spearman's test.

The validity of serum and BAL concentrations of KL‐6 used for differential diagnosis of ILD, including IPF versus non‐IPF patients, was assessed by areas under the receiver operating characteristic (ROC) curve. Sensitivity and specificity were calculated for cut‐offs of the different variables. The Youden index (J = max [sensitivity + specificity −1]) was used to establish the best cut‐offs. Statistical analysis was performed using GraphPad Prism 10 and Jamovi software.

## 3 Results

### 3.1 Study Population

A total of 158 patients (mean age ± standard deviation, 61.5 ± 13.7 years, 65 females) was included.

Table 1 shows clinical, demographic and immunological features of ILD patients stratified by diagnosis. No patients were on antifibrotic treatment at the time of sampling. Comparative analysis showed older patients with a significant male prevalence in IPF subgroups (83%) (*p* = 0.0012). Functional assessment showed mild restrictive impairment of lung volumes, associated with a moderate reduction in DLco percentages in IPF patients (Table 1). Alveolar immunological data were reported in Table 2 including BAL cellular patterns (macrophages, lymphocytes, neutrophils and eosinophils) and BAL lymphocyte subsets (CD3, CD4, CD8, CD19, CD16^+^CD56^+^).

**TABLE 1:** Clinical, demographic and immunological data of ILD patients stratified by diagnosis: Sarcoidosis, other ILDs, IPF and CTD‐ILD.

	Sarcoidosis (*n* = 74)	Other ILDs (*n* = 33)	IPF (*n* = 36)	CTD‐ILD (*n* = 15)
Age (M ± SD)	56.19 ± 12.4	65.52 ± 13.59^1^	73.05 ± 8.23^2^	63.92 ± 14.11
Gender (F/M)	33/41	15/18	6/30	11/4
FVC%	110.7 ± 15.14^3^	82.14 ± 11.32	77.25 ± 21.47	80.25 ± 11.03
FEV1%	97.75 ± 14.70^4^	81.36 ± 16.07	84.5 ± 24.17	85.5 ± 13.77
DLco%	82.32 ± 16.31^5^	68.64 ± 21.89	59.5 ± 16.91	73.67 ± 23.03
KL‐6 concentrations (U/mL)				
BAL‐FEIA	635.95 ± 691.99^6^	1256.35 ± 966	673.23 ± 602.97	652.58 ± 748.96
Serum‐FEIA	510.68 ± 261.08^7^	1155.63 ± 1144.4	1325.34 ± 637.51	996.31 ± 920.46
BAL‐CLEIA	626.75 ± 531.09	770.86 ± 612.10	319.75 ± 331.05^8^	490.45 ± 400.79
Serum‐CLEIA	567.24 ± 278.71^9^	1353.93 ± 1238.40	1658.5 ± 1145.48	1875.69 ± 2397.65

*Note:* All data is reported as mean ± standard deviation. Comparative analysis showed (1) younger patients with sarcoidosis than other ILDs (*p* = 0.0372) and (2) older patients with IPF than sarcoidosis (*p* < 0.0001). (3) Mean FVC percentages were higher in patients with sarcoidosis than in those with other ILDs (*p* = 0.0010), IPF (*p* = 0.0130) and CTD‐ILD (*p* = 0.05). (4) FEV1 percentages were higher in patients with sarcoidosis than other ILDs (*p* = 0.0162), while (5) DLco percentages were higher in patients with sarcoidosis than IPF (*p* = 0.0137). (6) BAL‐KL‐6‐FEIA concentrations were lower in patients with sarcoidosis than other ILDs (*p* = 0.0099) and (7) serum‐KL‐6‐FEIA concentrations were lower in patients with sarcoidosis than other ILDs and IPF (*p* = 0.0138 and *p* < 0.0001, respectively). (8) BAL‐KL‐6‐CLEIA concentrations were lower in patients with IPF than sarcoidosis (*p* = 0.0045) and other ILDs (0.0052). (9) Serum‐KL‐6‐CLEIA concentrations were lower in patients with sarcoidosis than other ILDs (*p* = 0.0009), IPF (*p* < 0.0001) and CTD‐ILD (*p* = 0.0079).

**TABLE 2:** BAL cell pattern including percentages of macrophages, lymphocytes, neutrophils and BAL immunophenotyping.

	Sarcoidosis (*n* = 74)	Other ILDs (*n* = 33)	IPF (*n* = 36)	CTD‐ILD (*n* = 15)
BAL cell pattern (%)				
Lymphocytes	20.49 ± 15.11^1^	10.95 ± 9.99	6.13 ± 3.31	6.88 ± 4.79
Macrophages	68 ± 20.13	63.15 ± 29.95	64.88 ± 26.13	65.75 ± 27.55
Neutrophils	9.47 ± 14.9	13.82 ± 23.68	23 ± 19.45^2^	21 ± 25.13
Eosinophils	1.1 ± 0.5	11.6 ± 10.1^3^	2.05 ± 0.3	8.6 ± 14.8
BAL immunophenotype				
CD16+CD56+ (NK cells)	3.08 ± 3.68	12.13 ± 12.07^4^	3.03 ± 0.93	2.16 ± 2.94
CD3 (T cells)	93.2 ± 6.25^6^	27.11 ± 41.17	92.4 ± 5.12	94.91 ± 3.23^5^
CD4 (Th cells)	69.05 ± 18.47	84.3 ± 18.89^7^	58.87 ± 12.44	54.38 ± 18.74
CD8 (Tc cells)	25.02 ± 16.44	53.83 ± 16.31^8^	33.27 ± 12.89	36.73 ± 16.95
CD19 (B cells)	1.37 ± 1.76	29.32 ± 18.84^9^	3.23 ± 4.71	1.73 ± 2.71

*Note:* Comparative analysis showed (1) higher alveolar lymphocytes in patients with sarcoidosis than in those with other ILDs (*p* = 0.0131), IPF (*p* = 0.0071) and CTD‐ILD (*p* = 0.0105). (2) Neutrophils was lower in patients with IPF than sarcoidosis (*p* = 0.0241), while (3) eosinophil percentages were higher in patients with other ILDs than sarcoidosis and IPF (*p* < 0.0001). (4) CD16+CD56+ cell percentages were lower in patients with sarcoidosis than other ILDs (*p* = 0.0035). (5) CD3 cell percentages were higher in patients with sarcoidosis than other ILDs (*p* < 0.0001), and (6) in patients with CTD‐ILD than other ILDs (*p* = 0.0094). (7) CD4 cell percentages were higher in patients with other ILDs than sarcoidosis (*p* = 0.0007), IPF (*p* = 0.0327) and CTD‐ILD (*p* = 0.0092). (8) CD8 cell percentages were lower in patients with sarcoidosis than other ILDs (*p* < 0.0001). (9) Mean CD19 cell percentages were higher in patients with other ILDs than sarcoidosis (*p* < 0.0001), IPF (*p* = 0.0210) and CTD‐ILD (*p* = 0.0021).

### 3.2 Analytical Validation of KL‐6

To analyse the reproducibility of CLEIA, KL‐6 values were tested three times in 10 fresh randomised serum and BAL samples, obtaining homogeneous results at each measurement (Table S1). After the same samples had been refrigerated (−80°C), KL‐6 concentrations were in line with those measured in fresh samples (Table S1).

Bronchoalveolar lavage and serum samples were assayed for KL‐6 by FEIA and CLEIA. Mean BAL concentrations of KL‐6 evaluated by FEIA were 784 ± 784 U/mL rather than CLEIA 587 ± 525 U/mL (*p* = 0.1801). Mean serum concentrations of KL‐6 evaluated by FEIA were 909 ± 786 U/mL rather than those evaluated by CLEIA 1158 ± 1236 U/mL (*p* = 0.0667).

The data showed a high quantitative correlation between the two tests (*p* < 0.0001). The Pearson correlation coefficient between FEIA and CLEIA measurements of KL‐6 in BAL was 0.707, and in serum 0.816.

Agreement between the two methods was quantified by the Bland–Altman test including all ILD patients. Measurements of KL‐6 in BAL by FEIA and CLEIA showed a mean bias of 178 (95% CI 82.7–273) with a lower limit of agreement of −915 (95% CI −1078 to −753) and an upper limit of 1271 (95% CI 1107.9–1433) (Figure S1a). In serum they showed a mean bias of −172 (95% CI −272 to −73) with a lower limit of agreement of −1303 (95% CI −1473 to −1132.5) and an upper limit of 958 (95% CI 788–1128.5) (Figure S1b). Stratifying patients according to diagnosis of sarcoidosis, CTD‐ILD, IPF and other‐ILD, Bland–Altman test was performed to confirm the agreement between the two methods FEIA versus CLEIA for detecting KL‐6 concentrations (Figure S2a–d).

Comparative analysis showed statistically significant differences between BAL and serum concentrations of KL‐6 measured by FEIA (*p* = 0.0106) and by CLEIA (*p* < 0.0001).

### 3.3 KL‐6 Concentrations in ILD Patients Measured by CLEIA

**FIGURE 2:**
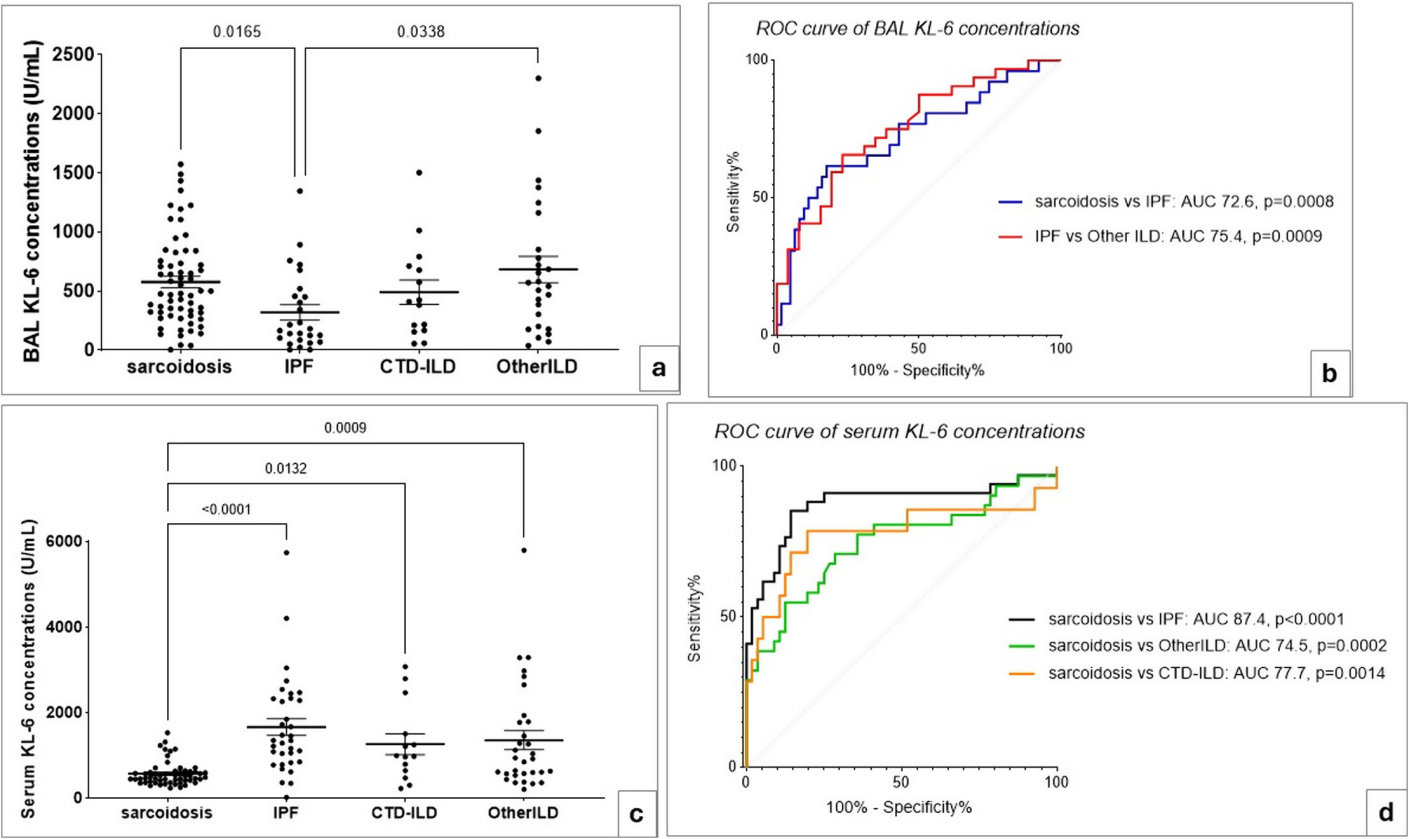
Comparative analysis and receiver operating characteristic (ROC) curve analysis using BAL and serum KL‐6 concentrations, detected by CLEIA method, in ILD patients stratified according to diagnosis. (a) Lower BAL‐KL‐6 concentrations in IPF patients than sarcoidosis (*p* = 0.0165) and other ILD (*p* = 0.0338) groups. (b) AUROC of 72.6% (*p* = 0.0008) identified BAL‐KL‐6 cut‐off value of 248.3 U/mL to distinguish IPF and sarcoidosis patients, while cut‐off value of 462.4 U/mL distinguished IPF and other ILDs with AUROC of 75.4% (*p* = 0.0009). (c) Lower serum KL‐6 concentrations in sarcoidosis than IPF (*p* < 0.0001), CTD‐ILD (*p* = 0.0132) and other ILD (*p* = 0.0009). (d) AUROC of 87.4% (*p* < 0.0001) identifies cut‐off of 1070 U/mL (SE 67.65% and SP 89.29%) to distinguish IPF and sarcoidosis, whereas 596.8 U/mL and 748.4 U/mL were the best to distinguish patients with sarcoidosis from those with other ILDs (AUROC 74.5%, *p* = 0.0002) and CTD‐ILD (AUROC 77.7%, *p* = 0.0014), respectively.

Bronchoalveolar lavage concentrations of KL‐6 were lower in patients with IPF than in those with sarcoidosis and other ILDs (*p* = 0.0165 and *p* = 0.0038, respectively) (Figure 2a). ROC analysis (figure sb) indicated that the best cut‐off values to distinguish patients with IPF and sarcoidosis were 248.3 U/mL (SE 61.54% and SP 82.54%), and 462.4 U/mL to distinguish patients with IPF and other ILDs (SE 65.63% and SP 76.92%).

Serum concentrations of KL‐6 (Figure 2c) were lower in patients with sarcoidosis than in those with IPF, CTD‐ILD and other ILDs (*p* < 0.0001, *p* = 0.0132 and *p* = 0.0009, respectively). The best cut‐off value (Figure 2d) to distinguish patients with IPF and sarcoidosis was 1070 U/mL (SE 67.65% and SP 89.29%), whereas 596.8 U/mL (SE 70.97% and SP 71.43%) and 748.4 U/mL (SE 71.43% and SP 85.71%) were the best to distinguish patients with sarcoidosis from those with other ILDs and CTD‐ILD, respectively.

**FIGURE 3:**
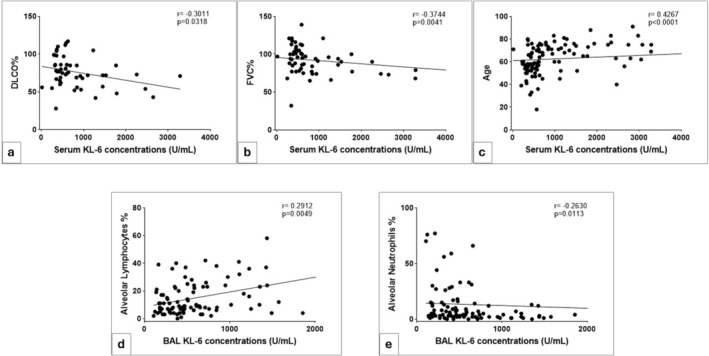
Spearman correlation tests between clinical features and serum and BAL CLEIA KL‐6 concentrations. Serum concentrations were inversely correlated with (a) DLCO% (*r* = −0.3011, *p* = 0.0318) and (b) FVC% (*r* = −0.3744, *p* = 0.0041), while they were directly correlated with (c) age (*r* = 0.4267, *p* < 0.0001). BAL KL‐6 concentrations were directly correlated with (d) alveolar lymphocyte percentages (*r* = 0.2912, *p* = 0.0049) and inversely correlated with (e) alveolar neutrophil percentages (*r* = −0.2630, *p* = 0.0113).

Figure 3 shows the main statistically significant correlations between CLEIA KL‐6 measurements and clinical features of ILD patients. The same results were obtained from spearman correlation tests between clinical features and serum and BAL KL‐6 concentrations assayed with FEIA method (Figure S3a–e).

### 3.4 KL‐6 Concentrations in Patients With Fibrotic ILDs Measured by CLEIA

**FIGURE 4:**
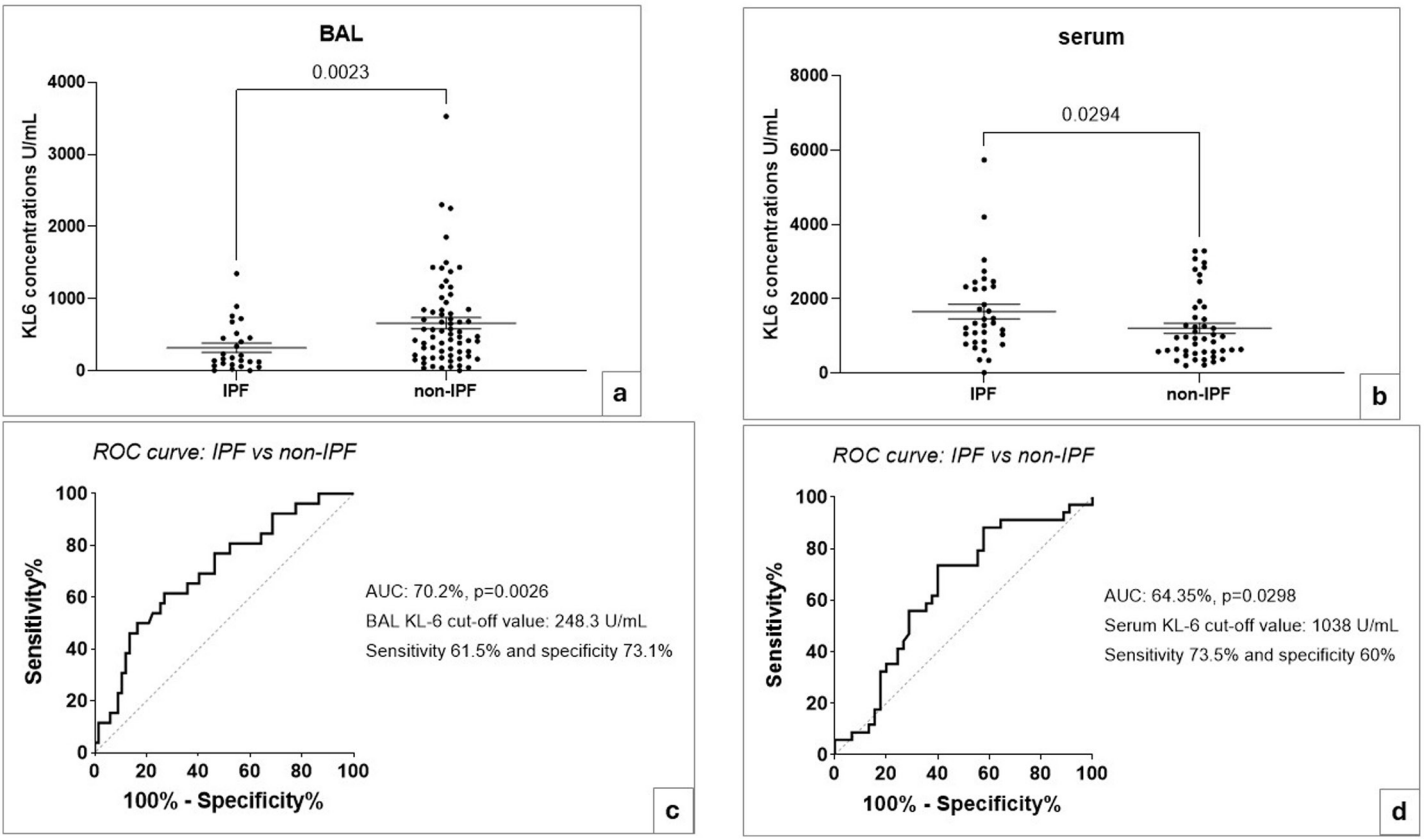
Comparative analysis and receiver operating characteristic (ROC) curve analysis stratifying patients according to IPF and non‐IPF using BAL and serum KL‐6 concentrations. BAL KL‐6 values (a) were lower in IPF than non‐IPF patients (*p* = 0.0023), while serum KL‐6 concentrations (b) were higher in IPF than non‐IPF patients (*p* = 0.0294). AUROC of 70.2% (*p* = 0.0026) identify the best BAL KL‐6 values (c) cut‐off value of 248.3 U/mL to distinguish IPF and non‐IPF patients. Serum KL‐6 cut‐off value (d) of 1038 U/mL serum allow to distinguish IPF and non‐IPF (AUROC 64.35%, *p* = 0.0298).

Patient groups were stratified according to IPF versus non‐IPF (including CTD‐ILD and other ILD). BAL concentrations of KL‐6 were lower in patients with IPF than in those with non‐IPF (*p* = 0.0023, Figure 4a), while serum concentrations of KL‐6 were higher in patients with IPF than in the non‐IPF group (*p* = 0.0294, Figure 4b).

Receiver operating characteristic curve analysis (Figure 4c,d) identified a cut‐off value of KL‐6 of 248.3 U/mL (SE 61.5% and SP 73%) in BAL and 1038 U/mL (SE 73.5% and SP 60%) in serum for distinguishing IPF and non‐IPF patients.

## 4 Discussion

Here we analysed KL‐6 concentrations in two biological fluids, BAL and serum, from patients presenting with signs and symptoms suggesting ILD. Two analytical methods, FEIA and CLEIA (Tosoh Biosciences), were compared in order to validate the diagnostic role of KL‐6 in fibrotic ILDs. We optimized the laboratory protocol for detecting KL‐6 in serum and BAL samples by FEIA and CLEIA. Before starting analysis, the reproducibility of CLEIA method was checked (Table S1).

A recent study analysed KL‐6 gene expression in BAL and serum by qRT‐PCR, highlighting higher serum expression in patients with progressive pulmonary fibrosis than with non‐progressive fibrosis. KL‐6 was much higher in serum than in BAL, but was not quantified.

The diagnostic division of Eidia Co. Ltd. (Tokyo, Japan) performed a wide‐ranging pioneer study on KL‐6 as a serum biomarker of lung disease [22, 23]. The findings led to development of an enzyme‐linked immunosorbent assay (ELISA) for determination of the absolute amount of KL‐6 in clinical samples.

A few years ago, a FEIA system (AIA‐360, Tosoh Biosciences) was developed to detect KL‐6 in serum samples and our research group performed an analytical verification and quality assessment of Tosoh AIA‐360 compared to Lumipulse G600II (CLEIA, Fujirebio). Both methods proved reliable and comparable for detecting KL‐6 in serum from hospitalized COVID‐19 and post‐COVID‐19 patients (2–5 months after discharge from hospital), highlighting the role of this protein in identifying fibrotic lung damage [15].

In the present study, we confirmed the role of KL‐6, detected with AIA‐360 (FEIA system, Tosoh Biosciences) and CL‐AIA (CLEIA system, Tosoh Biosciences) instruments, as a diagnostic and prognostic marker for fibrotic ILD patients. The agreement between the two methods was statistically significant for BAL as well as serum concentrations of KL‐6, even when ILD patients were stratified according to diagnosis (sarcoidosis, CTD‐ILD, IPF and other ILD). Our findings demonstrate that CLEIA and FEIA are comparable and reliable methods for detecting KL‐6 in serum and BAL samples. Pearson correlation showed a high score that indicates the similarities between FEIA and CLEIA for the detection of KL‐6 in BAL (0.707) and serum (0.816) samples. Recent study by Millan‐Billi et al. [24] reported a cut‐off value for serum KL‐6 greater than 465 U/mL with a good sensitivity and specificity to detect ILD. Several other studies have reported serum KL‐6 levels ranging from 1039.7 ± 823.7 U/mL to 2975 (450–5750) U/mL in IPF patients [25, 26]. Varied mean levels of serum KL‐6 in different studies may be due to geographical heterogeneity and difference in fibrotic lung proportion in included patients, as well as the different laboratory analytical methods (ELISA, FEIA, CLEIA, etc.).

Chemiluminescent enzyme immunoassay may therefore become a preferred and widely adopted method for further studies and its use may even be proposed in clinical practice. Rapidity (30 tests/h, time to first result approx. 15 min), automatic sample dilution (avoiding human error), 90‐day calibration stability for KL‐6 assays, minimum waste of reagents thanks to long expiry dates, optimization of biological sample volumes (~80 μL), continuous processing and digital data export are points in its favour.

Confirming our previous results [27, 28, 29], we stratified patients by diagnosis and we found BAL concentrations of KL‐6 lower in patients with IPF than sarcoidosis and other ILDs while serum concentrations of KL‐6 were higher in patients with IPF than sarcoidosis.

Alveolar lymphocytosis has been considered a key parameter in the diagnosis of sarcoidosis and our study cohort showed a direct correlation between alveolar lymphocytes and BAL concentrations of KL‐6, sustaining the role of the protein for detecting inflammatory alveolar damage.

The clinical parameters usually used to diagnose ILD patients were correlated with serum and BAL concentrations of KL‐6. At the time of diagnosis, the prognostic role of KL‐6 already reported in literature is further confirmed by our results, showing indirect correlations between serum measurements of KL‐6 and DLco% (*r* = −0.3011) and FVC% (*r* = −0.3744) highlighting patients with more advanced ILD, mainly those with IPF (a progressive fibrotic ILD).

Similarly, Qin et al. [30] also reported a negative correlation coefficient of −0.513 in their cohort between DLco and serum KL‐6 concentrations. Majewski et al. [31] studying various blood biomarkers in IPF patients found an inverse correlation between serum KL‐6 with FVC% at various time point (baseline *r* = −0.67, *p* < 0.001; 6 months *r* = −0.57, *p* < 0.01; 12 months *r* = −0.60, *p* < 0.001; 18 months *r* = −0.41, *p* < 0.05 and 24 months *r* = −0.50, *p* < 0.01). Their baseline rho coefficient was higher than our study, though the overall average correlation over all time points was comparable to ours.

Concerning the differential expression of KL‐6 in BAL and serum across different subgroups of ILDs, our findings confirmed the potential of this biomarker in discriminating between fibrotic and non‐fibrotic‐ILDs, especially considering the significantly lower values reported in the sarcoidosis patients. Interestingly, patients with sarcoidosis showed the higher values of KL‐6 in BAL, underscoring the epithelial damage ongoing due presumably to granulomatous inflammation, that however is not reflected in serum samplings. Fibrotic ILDs, particularly IPF reporting the lowest concentrations of KL‐6 values in BAL and conversely the highest on serum, showed a substantially opposite pattern of KL‐6 expression: this is probably related to the architectural distortion of lung parenchyma due to fibrogenesis and to the augmented permeability of alveolar‐capillary membrane induced by inflammation, which led to an increased release of KL‐6 in the bloodstream. Although our study contributes to the validation of an alternative laboratory method for KL‐6 routine analysis and the usefulness of KL‐6 in the early detection of specific patterns of lung damage, there are some limitations. First, healthy control subjects were not included since endoscopic procedures without diagnostic or therapeutic purposes are not allowed in Italy. Second, we didn't include patients with obstructive lung disorders (such as COPD or asthma) since bronchoscopic exams with sample samplings are admitted only if clinically justified, such as exacerbations, which may also alter KL‐6 assessment and not be feasible for the aims of the study.

From a clinical point of view, if validated on larger and more homogeneous cohorts, this biomarker may represent a precious tool for the differential diagnosis of early ILDs, that is frequently a real diagnostic challenge alone with radiological and clinical assessment.

## 5 Conclusion

Here we quantified KL‐6 concentrations by CLEIA, demonstrating the advantages of the method for routine assay of the biomarker including less time‐consuming, assay precision due to the auto‐sample dilution that minimizes the risk of human error and reproducibility of KL‐6 results in fresh and refrigerated serum and BAL samples that led to perform retrospective study. Moreover, we corroborate the role of KL‐6 for an accurate diagnosis of fibrotic lung damage in ILD patients at the time of presentation.

## Author Contributions

Conceptualization and designing research studies: Miriana d'Alessandro. Conducting experiments: Miriana d'Alessandro, Irene Paggi, Sara Gangi, Alfonso Brogna. Acquiring data: Miriana d'Alessandro, Tommaso Pianigiani, Giorgia Moriondo, Giusy Montuori, Giulio Natalello, Sofia Marrucci, Giulia Scioscia. Analyzing data: Miriana d'Alessandro. Visualization and supervision: Elena Bargagli, Paolo Cameli, Donato Lacedonia, Piera Soccio, Laura Bergantini. Writing the manuscript: all authors. All authors have read and agreed to the published version of the manuscript.

## Ethics Statement

The study was conducted in accordance with the Declaration of Helsinki, and approved by the Local Ethics Committee of Siena University Hospital (C.E.A.V.S.E.) (protocol code Markerlung 17431).

## Consent

Informed consent was obtained from all subjects involved in the study.

## Conflicts of Interest

The authors declare no conflicts of interest.

## Supporting information


Appendix S1

## Data Availability

The data that support the findings of this study are available on request from the corresponding author.
