# Supplementary material for: Diagnostic Performance of CLEIA Versus FEIA for KL‐6 Peripheral and Alveolar Concentrations in Fibrotic Interstitial Lung Diseases: A Multicentre Study
Source: J Clin Lab Anal. 2024 Sep 25;38(19-20):e25108. doi: 10.1002/jcla.25108 (PMC11520937; doi:10.1002/jcla.25108)
Supplement: Supplementary file 1 — Appendix S1 [file JCLA-38-e25108-s001.docx]

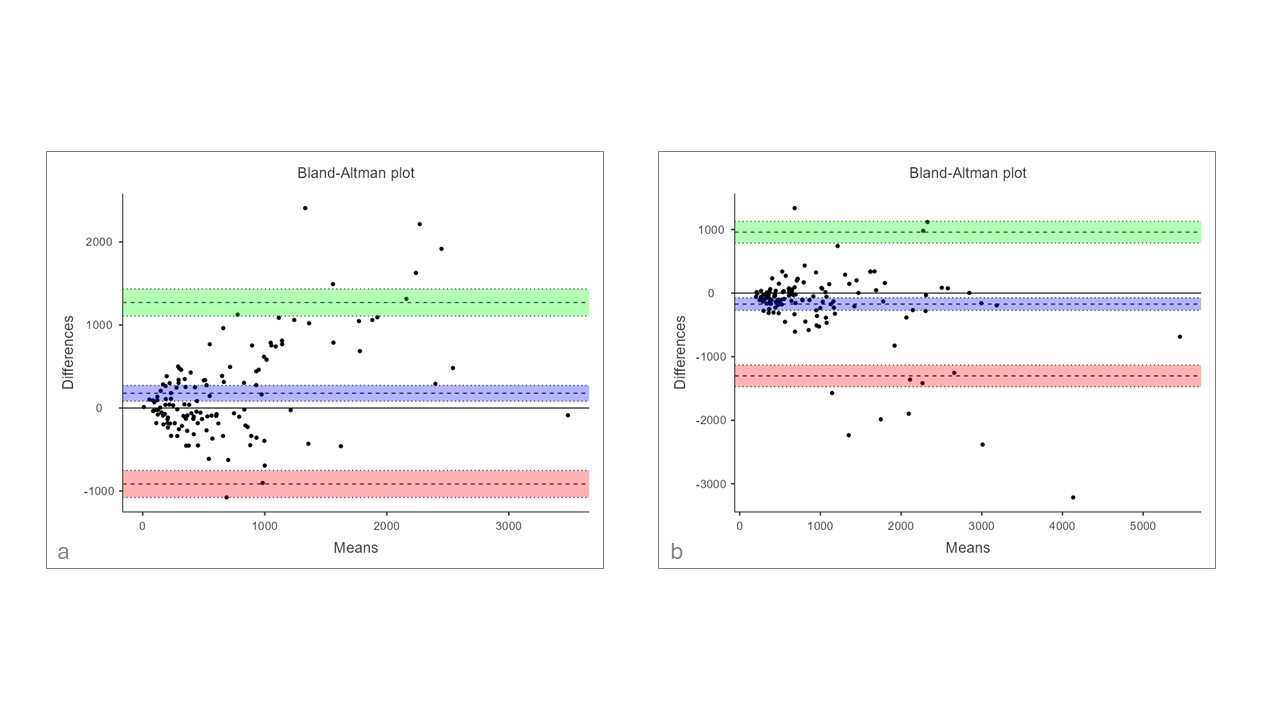


**Figure S1.** Blend-Altmann analysis performed to quantified agreement between the two methods, FEIA and CLEIA, according to BAL-KL-6 **(1a)** measurements of KL-6 with a mean bias of 178. Similarly, serum KL-6 **(1b)** concentrations obtained by the two methods were used for Blend-Altmann analysis resulting in a mean bias of -178.


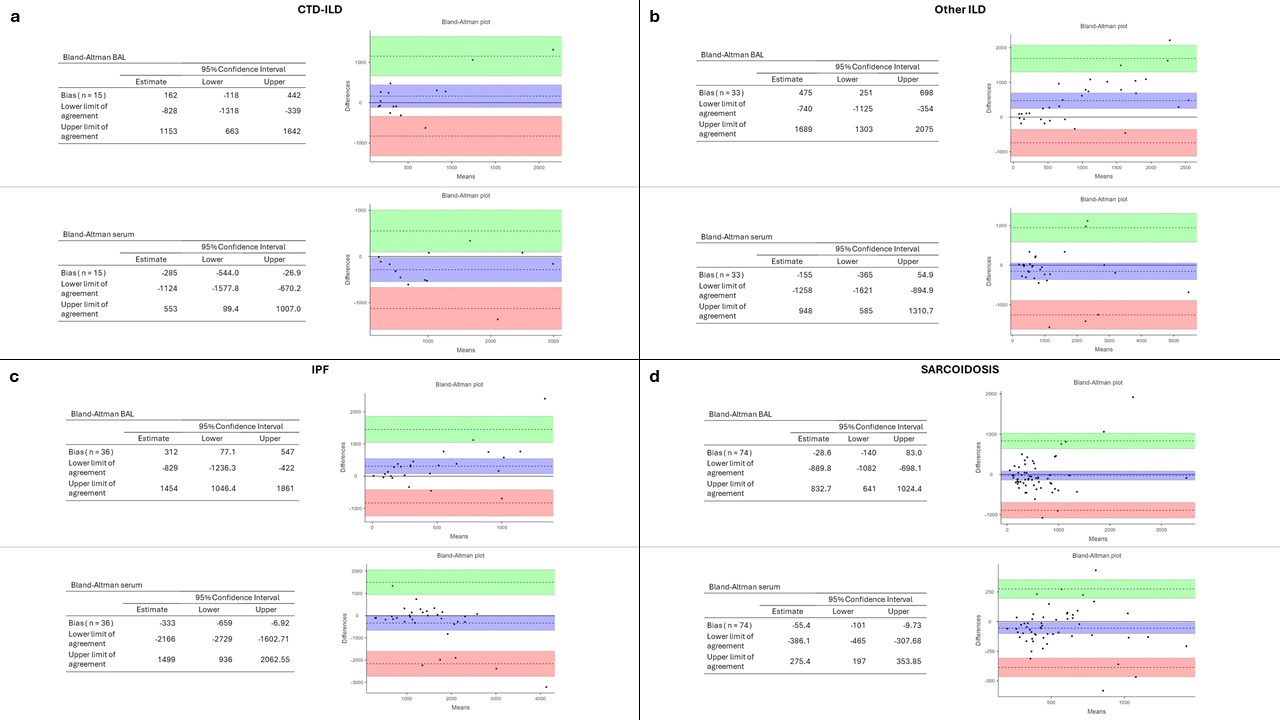


**Figure S2a-d**. Bland-Altman analysis to confirm the agreement between two methods, FEIA vs CLEIA, in BAL and serum samples for detecting KL-6 concentrations in patients stratifying according to diagnosis: s2.a, CTD-ILD; s2.b, other ILD; s2.c, IPF; s2.d, sarcoidosis. The lower and upper limit of agreement values were reported in the tables on the left of each figure (s2.a-s2.d).

Table S1. KL-6 measurements repeated three times in fresh and refrigerated randomised serum and BAL samples from 10 ILD patients.

| Record patinets | CLEIA BAL 1 (refrigerated) | CLEIA BAL 1 (fresh) | CLEIA BAL 2 (refrigerated) | CLEIA BAL 2 (fresh) | CLEIA BAL 3 (fresh) | CLEIA BAL 3 (refrigerated) | CLEIA SERUM 1 (refrigerated) | CLEIA SERUM 1 (fresh) | CLEIA SERUM 2 (refrigerated) | CLEIA SERUM 2 (fresh) | CLEIA SERUM 3 (fresh) | CLEIA SERUM 3 (refrigerated) |
| --- | --- | --- | --- | --- | --- | --- | --- | --- | --- | --- | --- | --- |
| 1 | 427.6 | 399.9 | 404.3 | 346.9 | 381.6 | 432.9 | 679.5 | 491.2 | 662.5 | 432.2 | 362.4 | 532.2 |
| 2 | 390.3 | 461.8 | 422.9 | 489.8 | 599.4 | 380.8 | 432.5 | 596.1 | 573.0 | 662.1 | 597.0 | 548.1 |
| 3 | 427.6 | 480.3 | 653.3 | 413.3 | 667.8 | 455.3 | 378.6 | 317.0 | 688.3 | 416.0 | 453.6 | 266.0 |
| 4 | 519.3 | 291.3 | 445.7 | 330.3 | 353.6 | 360.3 | 592.7 | 292.7 | 390.4 | 232.7 | 518.7 | 331.7 |
| 5 | 566.6 | 424.2 | 419.2 | 358.2 | 359.3 | 515.2 | 1137.2 | 1500.5 | 1013.4 | 1395.5 | 1305.0 | 1594.5 |
| 6 | 554.5 | 678.1 | 530.9 | 600.1 | 533.6 | 709.1 | 888.9 | 1245.7 | 1205.6 | 1128.7 | 995.2 | 1300.7 |
| 7 | 524.2 | 678.3 | 673.5 | 739.3 | 514.7 | 645.3 | 969.8 | 680.0 | 565.1 | 598.0 | 445.3 | 731.0 |
| 8 | 391.9 | 403.0 | 432.2 | 384.0 | 566.4 | 464.0 | 859.7 | 826.7 | 777.2 | 744.7 | 629.5 | 892.7 |
| 9 | 542.0 | 723.7 | 541.0 | 668.7 | 510.8 | 674.7 | 702.9 | 770.8 | 1102.6 | 703.8 | 597.5 | 809.8 |
| 10 | 823.0 | 893.4 | 637.1 | 812.4 | 663.8 | 922.4 | 740.3 | 1214.6 | 721.1 | 1110.6 | 723.6 | 1259.6 |


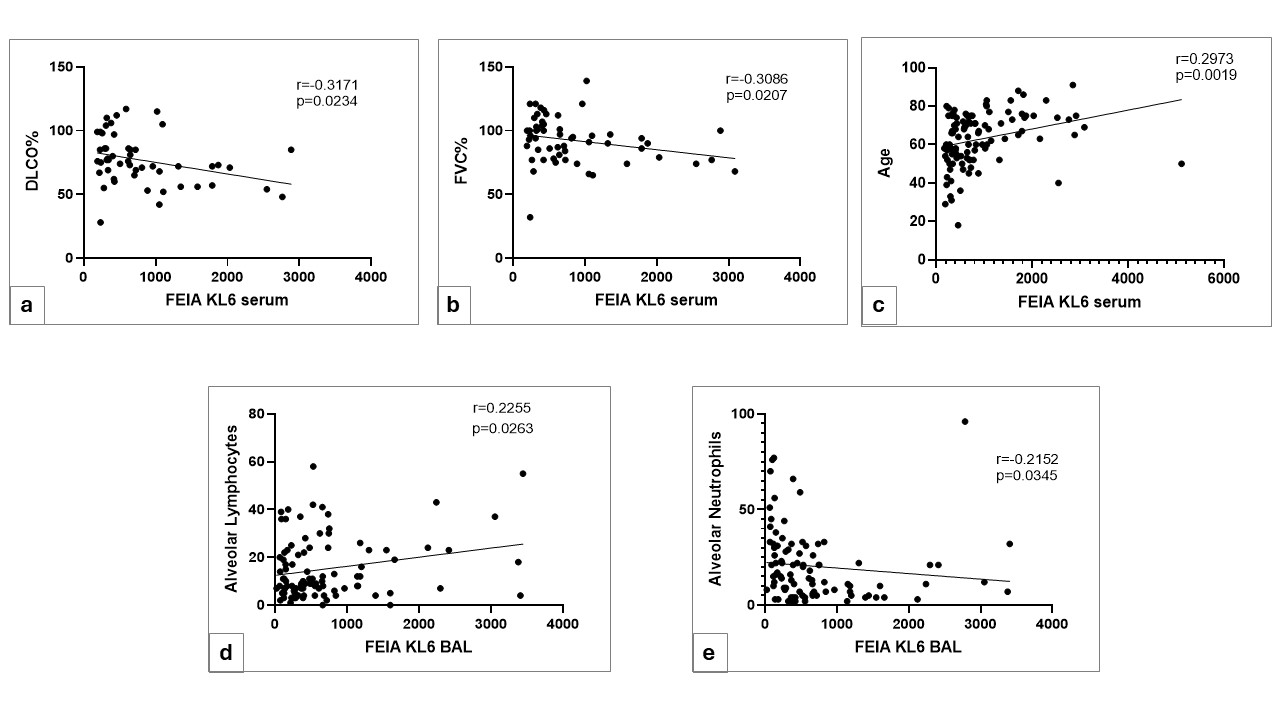


**Figure S3a-e.** Spearman correlation tests between clinical features and serum and BAL FEIA KL-6 concentrations. Serum concentrations were inversely correlated with **(s3a)** DLCO% (r=-0.3171, p=0.0234) and **(s3b)** FVC% (r=-0.3086, p=0.0207), while they were directly correlated with **(s3c)** age (r=0.2973, p=0.0019). BAL KL-6 concentrations were directly correlated with **(s3d)** alveolar lymphocyte percentages (r=0.2255, p=0.0263) and inversely correlated with **(s3e)** alveolar neutrophil percentages (r=-0.2152, p=0.0345).
